# Alternating sums of reciprocal generalized Fibonacci numbers

**DOI:** 10.1186/2193-1801-3-485

**Published:** 2014-08-29

**Authors:** Kantaphon Kuhapatanakul

**Affiliations:** Department of Mathematics, Faculty of Science, Kasetsart University, Bangkok, Thailand

**Keywords:** Fibonacci numbers, Reciprocal sums

## Abstract

**Abstract:**

Recently Holliday and Komatsu extended the results of Ohtsuka and Nakamura on reciprocal sums of Fibonacci numbers to reciprocal sums of generalized Fibonacci numbers. The aim of this work is to give similar results for the alternating sums of reciprocals of the generalized Fibonacci numbers with indices in arithmetic progression. Finally we note our generalizations of some results of Holliday and Komatsu.

**AMS Subject Classification:**

Primary 11B37; secondary 11B39

## Introduction

For a positive integer *p*, the generalized Fibonacci numbers *U*_*n*_ are defined for *n*≥0 by


If *p* = 1,2, then *U*_*n*_ are called the Fibonacci numbers *F*_*n*_ and Pell numbers *P*_*n*_, respectively. Also, if *p* is any variable *x*, then *U*_*n*_ are called Fibonacci polynomials *F*_*n*_(*x*).

(Ohtsuka and Nakamura 2008/2009) have found the formulas for the integer part of  and . (Holliday and Komatsu 2011) generalized these identities to the generalized Fibonacci numbers as follows
12

where ⌊·⌋ is the floor function.

Similar properties were investigated in several different ways; see (Wu and Zhang 2012; Wu and Zhang 2013; Zhang 2011). Recently, (Kuhapatanakul 2013) gave a similar formula (1) for alternating sums of reciprocal generalized Fibonacci numbers.

In this paper we consider the alternating sums of reciprocals of the generalized Fibonacci numbers with indices in arithmetic progression and evaluate the integer part to the reciprocals of these sums. That is, we derive and prove the formulas of the following forms


where *a*,*b* are non-negative integers with *b* < *a*. We also extend two identities (1) and (2) by replacing *U*_*k*_ with *U*_*ak*−*b*_.

## Main results

We begin with some identities of the generalized Fibonacci numbers whose will be used in the proofs of main theorem.

### **Lemma1**.

Let *n*,*r* be two integers with *n* > *r* > 0. Then

(i) *U*_*r*_*U*_*n*+1_ + *U*_*r*−1_*U*_*n*_ = *U*_*n*+*r*_.
(ii) *U*_*r*_*U*_*n*−1_ − *U*_*r*−1_*U*_*n*_= (−1)^*r*−1^*U*_*n*−*r*_.
(iii)

### *Proof*.

Every proof is done by induction and omitted.

### **Lemma2**.

Let *n*,*r* be two integers with *n* > *r* > 0. Then

(i)
(ii)

### *Proof*.

By using three identities of Lemma 1, we have


and


as desired.

We are now ready to verify our results.

### **Theorem1**.

Let *a*,*b* be two integers with 0 ≤ *b* < *a* and *f*(*n*) = *an*−*b*. Suppose *f*(*n*−1)>0 for all positive integer *n*. Then for *t* = 1,2 we have


### *Proof*.

Since the proofs of both cases *t* = 1 and *t* = 2 are quite similar, we only give a proof for *t* = 2. We see that *f*(*n*) > *a* for all *n*. By Lemma 2 (*ii*),


we get


Thus,


By applying the above inequality repeatedly, we obtain
3

In a similar way, we have


so


Repeating the above inequality, we obtain
4

Using Lemma 1(iii), we have


The numerator of the right hand side of above equation is positive if *f*(*n*−1) + *n* is even. Also, the numerator is negative if *f*(*n*−1) + *n* is odd.

Case I: If *f*(*n*−1) + *n* is even, then


so we obtain
5

Combining the (4) and (5), we get


it is equivalent to


where *f*(*n*−1) + *n* is even.

Case II: If *f*(*n*−1) + *n* is odd, then


We obtain
6

Combining the (3) and (6), we get


it is equivalent to


where *f*(*n*−1)+*n* is odd.

This completes the proof.

### **Remark1**.

- If *f*(*n*−1) + *n* = *a*(*n*−1) + *n* − *b* is even, then we have “both *a* and *b* are odd” or “ *a*,*b*,*n* are even” or “*a* is even and *b*,*n* are odd”.
- If *f*(*n*−1) + *n* = *a*(*n*−1) + *n* − *b* is odd, then we have “*a* is odd and *b* is even” or “ *a*,*b* are even and *n* is odd” or “ *a*,*n* are even and *b* is odd”.

Now we present some examples of Theorem 1 in the following corollary

### **Corollary1**

For a positive integer *n* > 1, we have

(i)
(ii)
(iii)
(iv)

Some examples for the Fibonacci numbers.

### **Example1**

For a positive integer *n* > 1, we have

(i)
(ii)

## Sums of reciprocals

We will obtain generalizations of the result of (Holliday and Komatsu 2011) on the reciprocal sums of *U*_*k*_ to the reciprocal sums of *U*_*ak*−*b*_.

### **Theorem2**.

Let *a*,*b* be two integers with 0 ≤ *b* < *a* and *f*(*n*) = *an* − *b*. Suppose *f*(*n*−1) ≥ 0 for all positive integer *n*. Then for *t* = 1,2 we have


### *Proof*.

We only give a proof for *t*=1 as that of *t*=2 is similar. Since *f*(*n*)≥*a* for all *n*, we get


Using Lemma 1 (*i*) and above inequality, we have


Thus, we obtain
7

Similarly, we can show that
8

On the other hand, we have


The numerator of the right hand side of above equation is positive if *f*(*n*−1) is odd. Also, the numerator is negative if *f*(*n*−1) is even.

If *f*(*n*−1) is odd, then we can verify that
9

and if *f*(*n*−1) is even, then we get
10

Combining the (7) and (9), we obtain


it is equivalent to


where *a*(*n*−1)−*b* is odd.

Combining the (8) and (10), we obtain


it is equivalent to


where *a*(*n*−1)−*b* is even.

### **Remark2**.

- If *f*(*n*−1) = *a*(*n*−1)−*b* is odd, then we have “*a* is even and *b* is odd” or “ *a*,*b*,*n* are odd” or “*a* is odd and *b*,*n* are even”.
- If *f*(*n*−1) = *a*(*n*−1)−*b* is even, then we have “both *a* and *b* are even” or “ *a*,*b* are odd and *n* is even” or “ *a*,*n* are odd and *b* is even”.

Since , if we take *a* = 1 in Theorem 2, we obtain the identities (1) and (2). Also, if we take *b* = 0, then we get the same results given in (Wu and Zhang 2013). In addition we have more results for *t* = 1,2 such as

(i)
(ii)
(iii)
(iv)
